# Short birth interval and associated factors among women of child bearing age in northern Ethiopia, 2016

**DOI:** 10.1186/s12905-019-0776-4

**Published:** 2019-07-02

**Authors:** Solomon Weldemariam Gebrehiwot, Gedamu Abera, Kidist Tesfay, Weyzer Tilahun

**Affiliations:** 1 Department of Midwifery, College of Health Sciences, Mekelle University, Mekelle, Tigray Ethiopia; 2 School of Nursing, College of Health Science, Mekelle University, Mekelle, Tigray Ethiopia

**Keywords:** Short birth interval, Child bearing women, Associated factors, Tigray, Ethiopia

## Abstract

**Background:**

Short birth interval is known to have a negative effect on perinatal, neonatal and child health outcomes. In Ethiopia, 29% of births are short birth intervals at less than 24 months. Even though optimum birth spacing is considered as an essential factor for the health of women and their children, to the best of the authors’ knowledge studies conducted on short birth interval are insufficient to inform policy makers. Therefore, the aim of this study was to assess short birth interval and associated factors among women of child bearing age in Tigray, Ethiopia.

**Methods:**

A community based cross-sectional study was conducted in Tselemti district among women of child bearing age from January 28 to February 28, 2016. Systematic sampling technique was used to select participants. Data were collected through face to face interviews and analyzed using SPSS version 20.0. Odds ratio along with 95% CI was computed to ascertain association between the outcome and predictor variables. A *p*-value of < 0.05 was considered as cut off point to assess significance of associations in the multivariable analysis.

**Results:**

The overall prevalence of short birth interval among women of child bearing age was 187 (23.3%). Sub-optimum breastfeeding (AOR = 7.01; 95% CI: 3.64, 13.46), non-use of contraceptive (AOR = 2.44; 95% CI: 1.55, 3.82), being Muslim (AOR = 2.02; 95% CI: 1.20, 3.40) and not having desire to had the last child (AOR = 3.63; 95% CI: 2.23, 5.91) were factors associated with short birth interval.

**Conclusion:**

Even though currently coverage of family planning use has increased, this study showed that short birth interval is still a concern for Ethiopian women due to factors such as: religion, suboptimum breastfeeding, unwanted pregnancy and non-use of contraceptives. Improving the accessibility and coverage of contraceptive use and involvement of religious leaders in family planning programs are essential strategies to be considered.

**Electronic supplementary material:**

The online version of this article (10.1186/s12905-019-0776-4) contains supplementary material, which is available to authorized users.

## Background

Birth spacing is an essential component of family planning (FP) and fertility control [1]. The 2005 World Health Organization (WHO) technical consultation group on birth spacing recommended a birth to conception interval of at least 24 months or a birth to birth interval of 33 or more months in two consecutive births [2]. Short birth interval (SBI) is known to have a negative effect on perinatal, neonatal and child health outcomes such as: preterm birth, still birth, intellectual disability and developmental delay [3–6]. On the other hand mothers may suffer from nutritional depletion, anemia, antepartum hemorrhage, cervical insufficiency, premature rupture of membranes and eclampsia [1, 4, 5]. In many studies, the maternal nutritional depletion hypothesis has been considered as a possible cause of SBI and adverse maternal and child outcomes [5, 7].

Currently, in developing countries more than 200 million women want either to space or limit pregnancies and yet they lack access to FP options [8]. Demographic Health Survey (DHS) studies show a high level of SBIs in many African countries (Rwanda: 20%; Uganda: 25.3%; Ethiopia: 20.4% and Cameroon: 21.3%) [9]. In Ethiopia, 29% of women had SBI of < 24 months [10]. Ethiopia has experienced a significant number of infant and neonatal mortality compared to the overall average rate of infant and neonatal mortality reported in Africa [11].

Subsequent births are likely in societies with sex preference in many African countries [1]. Such societal and cultural values contributed to high fertility with SBIs in the past and still persist as a challenge for the success of FP programs. In addition to this, socio-demographic factors, survival status of the index child, number of living children, employment, parity, breastfeeding duration and contraceptive use also contributed to high fertility rate with SBI [9, 12–17].

The Government of Ethiopia’s (GoE) strong investment in the health sector has contributed to significant progress over the last 15 years in the reduction of child mortality. For example, infant mortality was reduced from 166 in 2000 to 67 in 2016 and under-5 mortality from 97 in 2000 to 48 deaths per 1000 live births in 2016. Moreover, the use of contraceptive method increased from 6% in 2000 to 35% in 2016 and fertility rates decreased from 5.5 in 2000 to 4.6 children per woman in 2016 [8, 18]. Despite all these progress, Ethiopia is still behind the global FP and fertility targets. The country’s fertility and population growth of (2.5%) remain unacceptable and these make Ethiopia one of among the countries with high fertility rates in Africa [8]. Still, 22% of women in Ethiopia have unmet need for FP and 35% of contraceptive discontinuation rate [18].The Millennium Development Goal (MDG) target on maternal mortality (MMR) reduction was not achieved [19]. According to the DHS 2016, MMR remains at 412 per 100,000 live births for the country [18]. Furthermore, the change so far made in under-5 mortality reduction is stagnant in relation to neonatal mortality reduction [11]. Optimum child spacing is considered as an essential factor for the health of women and their children [13]. Hence, identifying factors which can affect birth interval of women is essential for countries burdened with high fertility and MMR like Ethiopia. However to the best of the authors’ knowledge, the available studies conducted on short birth interval in the country are insufficient to inform policy makers. Therefore, this study was conducted to assess SBI and underlying factors among women of child bearing age in Tselemti district, Northern Ethiopia.

## Methods

### Study design and area

A community based cross-sectional study was conducted in Tselemti district from January 28 to February 28, 2016. The district is located 1158 km North of Addis Ababa, the capital city of Ethiopia and 375 km North-West of Mekelle, the capital city of Tigray region. There were 25 kebeles, 16 health posts, 57 Health Extension Workers (HEWs), 6 Health centers and 1 primary hospital in Tselemti district according to the district health office (Unpublished report). According to 2007 central statistics, Tigray region has an estimated total population of 4,314,456, of which 2,189,603 of the population are females [20].

### Study population

The study population was women of child bearing age who experienced at least two successive births with the last birth occurring within the last five years prior to the data collection period. Women who had a history of twin and preterm births during the last delivery, those who have been living in the study district for less than six months and those who did not remember the exact birth date or did not have a birth certificate or immunization card for their child were excluded from the study. Moreover, women with history of any type of abortion in between the two successive births were excluded to obtain accurate measurement since the outcome variable of this study was measured from birth to birth in successive births.

### Sample size determination and sampling procedures

The sample size was calculated using a single population proportion formula [n = [Zα/2]^2^p (1-p)/d^2^] by considering 48% proportion of women who have SBIs from a previous study conducted in Tanzania to obtain a maximum sample size [21] with 95% (Zα/2 1.96) CI, 5% level of significance (α = 0.05), 5% of expected non-response rate and design effect of 2. The final sample size was 806. To identify study units, 10 of the 25 kebeles were selected randomly using lottery method. Then, 2–3 sub-kebeles (the smallest administrative unit) from each selected kebele were chosen again randomly using the lottery method. Household census and numbering was carried out in each selected sub-kebele to obtain a sampling frame (list of eligible participants) before the actual data collection period. PPS was used to allocate the sample size for each selected kebele, then to sub-kebeles based on their eligible population size. Systematic sampling technique was used to select the 806 participants at every k^th^ interval (k = 2, 3). The first participant was selected using lottery method. In cases when there were more than one eligible participant in the same household, one participant chosen using the lottery method.

#### Data collection tool and procedures

The questionnaire was developed by reviewing different published literatures and standardized to local context of application [3, 9, 13, 15–17] (Additional file 1). The major explanatory variables considered in this study were organized under six sections as follows. Section 1: Scio-demographic variables: age, ethnicity, residence, religion, education level and occupation status (including the husband’s). Section 2: Reproductive variables: parity, number of living children, birth date of children, duration of birth interval and knowledge on the disadvantage of SBI. Section 3: biological and behavioral characteristics: age at marriage, contraceptive use and duration of breastfeeding. Section 4: Prior child status: sex, survival status of the first child and a history of twin in the first delivery. Section 5: Intention of the women to have pregnancy: desire to have the last child and reasons for failing to achieve this desire. Section 6: Environmental factors: time taken to access nearby health facility, source of information and decision making status. In many studies these variables were speculated as potential factors for SBI which can explain the outcome variable. The variables were formulated and framed under the distal and proximate variables which were used by Bongaarts to determine fertility rate [22]. Factors such as contraceptive use, marriage and breastfeeding duration (one component of postpartum infecundability) were considered under proximate variables, whereas the rest of the variables were clustered under distal variables. From the foregoing, the following theoretical statement can be formulated: the proximate variables determine the length of birth interval directly, whereas the distal variables act upon biological and behavioral variables (proximate variables) which in turn influence the length of birth interval. The outcome variable of this study was SBI which was dichotomized in to “Yes = 1/No = 0” form. A birth that occurred at less than 33 months (24 months from birth to conception plus 9 months for pregnancy period) following a previous birth in two successive births was classified as having SBI, corresponding to WHO recommendation [2]. Birth interval was calculated as the time that elapsed between the birth date of the first child and the birth date of the second child. Only the interval between the 2 most recent consecutive births was measured to avoid recall bias. The actual birth interval was measured using either the respondents’ memory for the date of birth since majority of the participants were rural residents or birth certificate/immunization card for those who have. Data were collected by 15 HEWs through face to face interviews using a semi-structured and pretested questionnaire in a private room. Training for data collectors along with four supervisors was given for two days by the principal investigator. To ensure data quality, the questionnaire was first prepared in English and translated to Tigrigna (local language). The later version was translated back to English language, to ensure internal consistency. A pre-test was conducted among 10% (81 women) of eligible women in Adigebru kebele out of the study area but with population having similar socio-demographic characteristics. After the pretest had been conducted, modifications were made on some variables before the actual data collection was conducted. Data collectors and supervisors were local language speakers. On site supervision was carried out on a daily basis. The collected data were also checked daily for its consistency and completeness.

### Ethical issues

Ethical approval was obtained from the Institution Ethical Review Board (IERB) of Mekelle University, College of Health Sciences. Support letter was obtained from the Tigray Regional Health Bureau to Tselemti district health office and to respective kebeles. Written consent was obtained from study participants. Illiterate participants who were unable to write provided their consent using their finger print and this was considered as written consent.

### Data management and analysis

The data were checked manually for completeness, coded, entered, cleaned and analyzed using SPSS version 20.0 software. Descriptive analysis such as frequency, percent, median, mean and standard deviation were computed and the result was presented using text, tables and figures. Binary and multiple logistic regression analysis were performed. Variables with *p-*value of < 0.25 at bivariate analysis were considered for the multivariable analysis to control the effect of confounding variables. The goodness of fit was tested by Hosmer-Lemeshow statistic and variables with *p-*value greater than 0.05 were fitted to the multivariable model. Odds ratio along with 95% confidence interval (CI) was computed to ascertain the strength of association between the explanatory and outcome variables. Statistical test at *p-*value of < 0.05 were considered as cut off point to assess significance of associations in the multivariable analysis.

## Result

### Socio-demographic characteristics

A total of 806 eligible participants were sampled, of these 803 responded completely resulting in a response rate of 99.6%. Majority of the participants 845 (80.3%) were married and 158 (19.7%) widowed/divorced. The mean age of the women at interview was 30.8 (SD + 22.8) years. Majority of the participants were Orthodox Christians 684 (85.2%) and the rest were Muslims. Table 1 shows the remaining socio-demographic characteristics of the participants.

**Table 1:** Socio-demographic characteristics among child bearing women in Tselemti district, Tigray, Ethiopia, 2016

Variables	Frequency (*N* = 365)	Percent (%)
Age		
20–24	85	10.6
25–29	254	31.6
30–34	245	30.5
35–39	164	20.4
40–50	55	6.8
Ethnicity		
Tigray	707	88
Amhara	96	12
Residence		
Urban	289	36
Rural	514	64
Educational status		
Illiterate	525	65.3
Elementary (1–8)	171	21.3
Secondary & above	107	13.3
Occupational status		
House wife	100	27.4
Farmer	202	55.3
Civil servant	20	5.5
Business women	28	7.7
Daily laborer	15	4.1
Husband’s occupation		
Government employee	92	14
Farmer	310	48
Merchant	180	28
Daily laborer	63	10
Husband’s education(N 645)		
No formal education	329	51
Elementary	175	27
Secondary & above	141	22

### Reproductive, behavioral and child status characteristics of the study participants

The median duration of breast feeding was 24 months (IQR = 6). One hundred ninety four (24.2%) participants were married at the age of below 15 years old, 384 (47.8%) between 15 and 17 and the rest were at 18+ years. Table 2 shows the detail reproductive and other characteristics.

**Table 2:** Reproductive, behavioral and child status characteristics among women of child bearing age in Tselemti district, Tigray, Ethiopia, 2016

Variables(*N* = 803)	Frequency	Percent
Parity		
1–2	221	27.5
3	206	25.7
4+	376	46.8
Birth interval in months		
< 33	187	23.3
33–59	403	50.2
60+	213	26.5
History of FP use		
Yes	618	77
No	185	23
Duration of breastfeeding in the index child(months)		
0–12	59	7.3
13–23	110	13.7
24+	634	79
No. of living children		
0–1	267	33.3
2	204	25.4
3	174	21.7
4+	158	19.7
Knowledge on short birth interval		
Yes	714	88.9
No	89	11.1
History of multiple births in index child		
Yes	32	4
No	771	96
Age of the woman at birth		
< 19	482	60
20+	321	40
Sex of the index child		
Male	428	53.3
Female	375	46.7
Survival of index child before the conception of the last child		
Yes	771	96
No	32	4

### Intention of participants to become pregnant and stated reasons

**Fig. 1:**
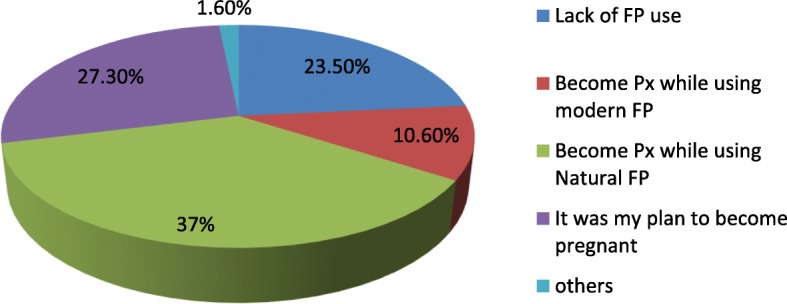
Stated reasons by women of child bearing age why they had short birth interval in Tselemti district, Tigray, Ethiopia, 2016

About 665 (82.8%) of respondents had intention to have the last child before conception and the rest (17.2%) were wanted to limit the number of children. Among those who had the intention to have the last child, 200 (30.1%) of them did not have a specific plan when to have that child, 14 (2.1%) wanted to have a child within 24 months, 92 (13.8%) wanted after 24 months but before 36 months and 359 (54%) after 36 months or more. Among the women who had plan, 144 (21.6%) of them got pregnant prior to their plan. The reasons were stated as husband pressure 66 (45.8%), death of the index child 15 (10.4%), failure of contraceptive methods 27.6% (natural method 34 (23.6%) and modern method 6 (4%)) and non-use of contraceptive 23 (16%). One hundred thirty eight (17.2%) women became pregnant while wanting to limit child bearing as a result of contraceptive failure 36 (26%), non-use of contraceptive 54 (39%), wrong perception that a woman would not be able to conceive yet because her menses has not resumed 46 (33.3%) and husband pressure for more children 2(1.7%). Figure 1 shows the reasons stated by women who had SBIs.

### Decision making autonomy and source of information

Concerning women’ decision making autonomy with regards to reproductive health care service utilization, 157 (19.6%) of them usually made decisions themselves, whereas 515 (64.1%) made decisions jointly with their husbands. However, in 131 (16.3%) of women the decision was made by their husbands. Regarding the source of information for their health, 20 (2.4%) of participants got from mass media, 433 (54%) from health professionals, 341 (42.4%) from combination of health professionals and mass media and the rest had no access to information at all. The median time taken from home to the nearby public health centers on foot was 30 min (IQR = 15).

### Practice of birth spacing

The overall short birth interval (< 33 months) among respondents was 187 (23.3%). Of these, 48 (6%) respondents had very SBI which is less than 24 months. In addition, 403 (50.2%) of participants had a range of Birth interval (BI) 33–59 months and 213 (26.5%) had 60+ months. The median duration of actual BI among participants was 44 (IQR = 29) and yet their preference BI was 40 months (IQR = 12).

### Factors associated with short birth interval

Cross tabulation and logistic regression analysis was carried out to assess the association between independent variables and SBI. Hence, age, religion, decision making status, distance, history of contraceptive use, number of living children, type of pregnancy(wanted/unwanted), duration of breastfeeding, survival status of the first child and knowledge of participants on SBI were found to be significantly associated with the outcome variable in bivariate analysis. These variables were adjusted to control confounding effect in multivariable logistic regression analysis and 4 of the factors had significant association with the outcome variable. Table 3 **shows** the detail of the regression result. Those who Muslims were about 2.02 times more likely to have SBI compared to Orthodox Christians **(AOR = 2.02; 95% CI: 1.20, 3.40)**. The odds of SBI among women whose breastfeeding duration < 12 months in the first child was 7 compared to women who had duration of breastfeeding 24+ months **(AOR = 7.01; 95% CI: 3.64, 13.46)**. Women who had no desire to have the last child were 3 times more likely to have SBI when compared to their counter parts **(AOR = 3.63; 95% CI: 2.23, 5.91)**.The odds of SBI among women who did not use contraceptive method before the last child was 2.4 as compared to users **(AOR = 2.44; 95% CI: 1.55, 3.82)**.

**Table 3:** Factors associated with short birth interval among childbearing women in Tselemti district, Tigray, Ethiopia, 2016

Variables (N = 803)	Short birth interval		COR (CI: 95%)	AOR (CI: 95%)	*p*-Value for AO*R*
	No n (%)	Yes n (%)			
Age at interview					
20–24	55 (64.7)	30 (35.3)	2.78(1.20, 6.46)	2.29(0.68, 7.64)	
25–29	192 (75.6)	62 (24.4)	1.65(0.76, 3.56)	2.04(0.68, 6.10)	
30–34	191 (78.0)	54(22.0)	1.44(0.66, 3.13)	2.19(0.77, 6.23)	
35–39	132 (80.5)	32 (19.5)	1.23(0.55, 2.79)	2.37(0.84, 6.65)	
40+	46 (83.6)	9(16.4)	1	1	
Religion					
Orthodox	541 (79.1)	143 (20.9)	1	1	
Muslim	75 (63.0)	44 (37.0)	2.21(1.46, 3.36)	2.02 (1.20, 3.40)^*^	0.008
Decision making					
Husband	86 (65.6)	45 (34.4)	1.92(1.26, 2.92)	1.64(0.98, 2.75)	
Self	125 (79.6)	32 (20.4)	0.943(0.60, 1.46)	0.83(0.48, 1.44)	
Both with discussion	405 (78.6)	110 (21.4)	1	1	
Time taken to health center/min					
< 30	472 (77.1)	140 (22.9)	1	1	
31–60	99 (80.5)	24 (19.5)	0.81 (0.50, 1.32)	0.61(0.33, 1.12)	
61–120	26 (61.9)	16 (38.1)	2.07(1.08, 3.97)	2.23(0.98, 5.08)	
> 120	19 (73.1)	7 (26.9)	1.24 (0.51, 3.01)	1.10(0.34, 3.47)	
FP use before last child					
Yes	516 (83.5)	102 (16.5)	1	1	
No	100 (54.1)	85 (45.9)	4.30(3.00, 6.15)	2.44(1.55, 3.82)^**^	0.001
No. of living children before the index child	184 (68.9)	83 (31.1)	1.70 (1.07, 2.71)	1.68 (0.82, 3.45)	
< 1	172 (84.3)	32 (15.7)	0.70 (0.41, 1.20)	0.64 (0.30, 1.34)	
2	135 (77.6)	39 (22.4)	1.09 (0.64, 1.84)	1.14 (0.57, 2.28)	
3	125 (79.1)	33 (20.9)	1	1	
4+					
Wanted pregnancy					
Yes	555 (83.5)	110 (16.5)	1	1	
No	61 (44.2)	77 (55.8)	6.36(4.29, 9.43)	3.63 (2.23, 5.91)^**^	0.001
Duration of BF in the index child (months)					
0–12	22 (37.3)	37 (62.7)	10.86(6.11,19.30)	7.01(3.64, 13.46)^**^	0.001
13–23	45 (40.9)	65 (59.1)	9.32(5.98,14.53)	6.12(3.68, 10.17)^**^	0.001
> 24	549 (86.6)	85 (13.4)	1	1	
Survival status of the index child					
Alive	596 (77.3)	175 (22.7)	1	1	
Dead	20 (62.5)	12 (37.5)	2.04(0.98,4.26)	1.38(0.58, 3.31)	
Knowledge on short birth interval					
Yes	558 (78.2)	156 (21.8)	1	1	
No	58 (65.2)	31 (34.8)	1.91(1.19, 3.06)	1.67(0.92, 3.03)	

COR = Crud odd ratio, AOR = Adjusted odd ratio

## Discussion

The overall prevalence of SBI (< 33 months) among the child bearing women was 187 (23.3%). This shows that nearly one fourth of the women are still experiencing SBIs with its consequences. This finding is consistent with the findings from Bangladesh and Iran, where about 24.6 and 28.5% of women had SBI respectively [8, 23]. In fact, this is a significant progress when compared to previous studies conducted in different parts of Africa. For example, 57.6% of women from Southern Ethiopia, 68% from developing countries in general and 48.4% from Tanzania reported SBIs [13, 21, 24]. This discrepancy could be due to the intervention that has been undertaken on FP utilization by GoE. This is evidenced by increment of FP coverage from 6% in to 35% and fertility reduction from 5.5 in to 4.6 [8, 18]. During the past two decades, the GoE has made significant progress in contraceptive access and utilization. However, there are still many women with unmet need for FP methods and women who depend on short-term contraceptives [8]. The contribution of the HEWs in promoting contraceptive use behavior and increasing availability of contraceptive to all Ethiopian women is indispensible [19]. Moreover, the desire to have more children which is rooted in the community has been changed due to economic concerns and changes in life style [25]. The finding of this study also shows that more than three fourth (77%) of study participants were contraceptive users preceding the conception of the last child. The median BI of this study was 44 months. This is near similar with the study conducted in Iran which was 39 months [23].

Muslim participants were about 2 times more likely to have SBI when compared to Orthodox Christians. Several studies agree with this finding. For example, studies done in Bangladesh and Nigeria confirm these finding [3, 26]. Another study from Ethiopia also reported that Christians tend to space births longer when compared to Muslims although it was not statistically significant [16]. This could be due to non-use of contraceptive methods among Muslim followers. A study conducted in Bahir-Dar reported 63.9% of Orthodox Christian followers versus 36.1% of Muslim followers were using modern contraceptives [27]. Moreover, it was also statistically significant in that study. Our study also shows the difference between Orthodox Christians and Muslims in use of contraceptive methods, in which 78% of Christians versus 69.7% of Muslims were ever used modern contraceptives before the conception of the last child. In fact, use of birth control methods has been established throughout the stages of Muslim development in the form of natural methods in general and withdrawal techniques/coitus interruption in particular [28]. Moreover, the Quran neither prohibits birth control nor approved a husband or wife to space pregnancy or limit their number. However, some Muslims perceive it as a concept which is totally against the principles of Islam [28, 29].

Participants who had never used contraceptive methods preceding the conception of the last child were more likely at risk of having SBIs compared to their counterparts. This is obviously clear that the purpose of contraceptive method is either to limit or space births [30]. Studies conducted in Haiti and Sudan reported that use of contraception was statistically significant with having optimum BI [31, 32]. Other studies from Southern and Northern part of Ethiopia also reported that non-use of contraceptive method was a significant factor associated with SBI [13, 15, 33].

Duration of breast feeding was a significant factor in this study. This is consistent with the study done in Southern part of Ethiopia [13]. It has long been recognized that women who breastfeed their children for longer duration have a longer period of amenorrhea which results in post partum infecundability when compared to their counterparts [34]. During breastfeeding the receptors in the breast nipple are stimulated and this initiates a signal to the hypothalamus: a nerve center in the brain which in turn signals the pituitary gland which inhibits ovulation by reducing the release of gonadotrophic hormone needed for ovulation which results in post partum amenorrhea [35]. The likelihood of having SBI among women who had no desire to have the last child is in line with studies conducted in the United State of America and Ethiopia [36, 37]. It is known that non-use of contraceptives and contraceptive failure are among the reasons for unintended pregnancy [37]. Adequate training for data collectors and pretesting of tools in population with similar socio-economic status could be the strength of this study.

### Limitation

This study was not without limitation. For example, successive BIs and breastfeeding duration were calculated based on the women recall for most participants; this may result in recall and reporting biases. Since this study was conducted in one district, the findings might not be generalizable to the entire region. The cross-sectional nature of the study does not allow for causality inferences. In addition, data for history of menses resumption and sexual behavior after delivery were not assessed even though these variables are important in the analysis of post-partum amenorrhea and post-partum infecundability which can affect birth interval of the women.

## Conclusion and recommendation

Even though currently coverage of FP use has increased, this study shows that SBI is still a concern for Ethiopian women as a result of religion, suboptimum breastfeeding, unwanted pregnancy and non-use of contraceptive factors. Currently, SBI has decreased as compared to the previous studies in Ethiopia. However, nearly one fourth of women are still at risk for adverse maternal and child outcomes resulted from SBI. These findings highlight the need to address these gaps. Therefore, the following recommendations are forwarded to midwives and other stakeholders to strengthen the FP service in terms of quality and utilization. Corresponding with increasing levels of contraceptive use, they should also encourage optimum breastfeeding and involvement of religious leaders in FP programs.

## Additional file

Additional file 1: English version questionnaire used in this study. (DOCX 25 kb)

## Data Availability

The datasets used and/or analyzed during the current study are available from the corresponding author upon reasonable request.

## Abbreviations

AOR: Adjusted odd ratio
BI: Birth interval
CI: Confidence interval
DHS: Demographic health survey
FP: Family planning
GoE: Government of Ethiopia
HEW: Health extension workers
MMR: Maternal mortality ratio
SBIs: Short birth intervals
SD: Standard deviation
WHO: World health organization
