# Supplementary material for: Short birth interval and associated factors among women of child bearing age in northern Ethiopia, 2016
Source: BMC Womens Health. 2019 Jul 2;19:85. doi: 10.1186/s12905-019-0776-4 (PMC6604155; doi:10.1186/s12905-019-0776-4)
Supplement: Supplementary file 1 — English version questionnaire used in this study. (DOCX 25 kb) [file 12905_2019_776_MOESM1_ESM.docx]

ANNEXES

**Annex I**: Information sheet

**Title of the project:** Short Birth Interval and Associated Factors among Child Bearing Woman in Tselemte district Northwest Tigray, Ethiopia.

**Principal investigator:** Solomon Weldemariam

**Co-investigator**: 1. Gedamu Abera

2. Kidist Tesfay

3. Weyzer Tilahun

**Sponsoring Institution**: Mekelle University

Greeting:

Hello, My name is_____________________. I am here today to collect data on the title mentioned above being conducted by Mr. Solomon weldemariam from Mekelle University, College of Health Science Department of Midwifery. The objective of this study is to assess the prevalence of short birth interval and associated factors here in Tselemtidistrict. I request you to take part in this study and to respond genuinely.

Your cooperation and willingness is greatly helpful in identifying the level and factors associated with short birth interval in this district. The study will be conducted through face to face interviews in a privacy and you are being asked for a little of your time, about 20-25 min, to help us in this study. All information given by you will be kept strictly confidential. Your name will not be written in this form and will never be used in connection with any information you tell us and the result will be disseminated in aggregated form. This study will be contributed to improve the health of mothers in this district as well as at nation level by providing information to policy makers by improving the health delivery system. There is no possible risk associated with participating in this study except, the time you spent for responding to the questionnaire. There is no any compensation and incentives with money or material directly for your participation in this study but you will be benefited from the quality service which expected to be improved by government based on this findings. Your participation is voluntary and you are not obliged to answer any question you do not wish to answer. If you feel discomfort with the question, it is your right to interrupt at any time you want. If you have questions regarding this study or would like to be informed of the results after its completion, please feel free to contact the principal investigator.

Address of the principal investigator: Solomon Weldemariam

Cell phone: +251 913941845/0943638866, E-mail: mikiass1708@gmail.com

You can use Mekelle University CHS Institutional Ethical Review Board (IERB) with the following address. Cell phone: +2510914703261/0910349281

Are you willing to participate in this study?

1. Yes - ………………………….. Continue to the next page

2. No- ……………Thank you……………… Skip to the next participant

**Annex II: Consent form**

In signing this document, I am giving my consent to participate in the study titled “Short birth interval and associated factors among child bearing women in Tselemtidistrict”. I have been informed that the purpose of this study and I have understood that participation in this study is entirely voluntarily. I have been told that my answers to the questions will not be given to anyone else and no reports of this study ever identify me in any way. I have also been informed that my participation or non-participation or my refusal to answer questions will have no effect on me. I understood that participation in this study does not involve risks. I understood that I will be benefited from this study indirectly. I understood that Solomon Weldemariam is the contact person if I have questions about the study or about my rights as a study participant.

Respondent’s signature_________________________________

If no, skip to the next participant

Date of interview: _______________ Time started: _______ Time finished: _________

Interviewer Name_________________________Signature___________Date____________

Supervisor’s name ________________ signature ________

Results of interview questionnaire

1. Completed

2. Refused

3. Partially completed

**Annex III: English Version Questionnaire**

Mekelle University, College of Health Sciences, Department of Midwifery Survey Questionnaire for assessment of Short Birth Interval and Associated factors among Child Bearing Woman in Tselemtidistrict North West Tigray, Ethiopia: A cross-sectional study.

002. Questionnaire ID number_________

003. Address: kebele ______________

Note: Encircle from the given option and write if any other idea or answer is given

**Section I. Socio-demographic characteristics of participants**

| No | **Questions** | **Response** | **Skip pattern** |
| --- | --- | --- | --- |
| 101 | Age in completed years | **___________years** |  |
| 102 | Ethnicity | 1. Tigray  2. Amhara  3.Other______ (specify) |  |
| 103 | Marital status | 1. Married  2. Single  3. Divorced  4. widow  5. Separated  6. cohabitated |  |
| 104 | Residence | 1.Urban  2.Rural |  |
| 105 | Religion | 1. Orthodox  2. Muslim  4. Protestant  5.Others(specify)____________ |  |
| 106 | Maternal education | 1. Un able to read and write  2. Informal learner able to read and write  3. Elementary school [1-8]  4. high school (9-10]  5. Diploma and above |  |
| 107 | Maternal occupation | 1. Housewife  2. Farmer  3. Civil servant  4. Business woman  5. other(specify)_________ |  |
| 108 | Husband’s education | 1. Un able to read and write  2. Informal learner able to read and write  3. Primary school (1-8)  4. high school(9-10)  5. Diploma and above |  |
| 109 | Husband’s occupation | 1. Civil servant  2. Farmer  3. merchant  4. Others(specify)---------- |  |

**Section II: Reproductive and knowledge characteristics**

| No | **Questions** | **Response** | **Skip** |
| --- | --- | --- | --- |
| ***Reproductive characteristics*** | | | |
| 201 | Birth date of the index child in E.C? | ___/___/______E.C |  |
| 202 | Birth date of the last child in E.C? | ___/___/______E.C |  |
| 203 | Duration of birth interval in months*?(Calculate using the above 2 Q 201 & 202)* | __________months |  |
| 204 | Parity(Total No of births) | 1. No of children alive_________  2. No of children died:_________  3. No of still birth____________ |  |
| 205 | Write the sum of Q 204 | ____________(total) |  |
| 206 | Number of living children before the last child? | _____________ |  |
| ***Maternal Knowledge on disadvantage of short birth interval*** | | | |
| 207 | Have you ever heard about optimum or short birth interval between live births? | 1Yes  2.No | If no skip to Q 212 |
| 208 | If yes, could you mention the optimum birth interval between two successive births? (*you can ask eithr the duration between birth or between birth and pregnancy then put birth interval in births interval*) | _________months |  |
| 209 | What are the disadvantages of short birth interval on you? (*multiple answer possible but do not mention the alternatives*) | 1. Increases nutritional deficiency  2. Increases morbidity during pregnancy and delivery  3.Others_______________________(specify)  4. I do not know |  |
| 210 | What are the disadvantages of short birth interval on your children? (*multiple answer possible but do not mention the alternatives*) | 1. Increases under-five mortality  2. Increases low birth weight  3. Increases preterm delivery  4. Increases still birth  5. Increases malnutrition  6. I do not know |  |
| 211 | How can you prevent short birth interval? (*multiple answer possible but do not mention the alternatives*) | 1. By using modern family planning methods  2. By using natural family planning methods  4.others__________________(specify)  5. I do not know |  |
| ***Biological and Behavioral characteristics*** | | | |
| 301 | Age at marriage(In completed years) | _________Years old |  |
| 302 | Age at first birth(In completed years) | _________years old |  |
| 303 | Contraceptive use before the conception of the last child? | 1.Yes  2. No |  |
| 304 | Duration of breast feeding for the index child/preceding the last child in months? | ____________months |  |
| ***Characteristics of the Previous Child*** | | | |
| 401 | Sex of the index child(The child before the last child) | 1. Male  2. Female |  |
| 402 | Survival of the index child before the conception of the last child? (The child before the last child) | 1. Alive  2. Dead |  |
| 403 | Multiple births history in index child(The child before the last child) | 1.Yes  2. No |  |
|  | **Intention of respondents to become pregnant before the last child** | | |
| 501 | Did you have the desire to have the last child? | 1. Yes  2. No | If yes skip to Q **221** |
| 502 | If **none**the answer for **Q 219** what were the reason for failing to limit that pregnancy | 1. Lack of family planning use  2. I become pregnant while I were using modern family planning methods  3. I become pregnant while I were using traditional family planning methods  4. others_____________________(specify) |  |
| 503 | If yes the answer to **Q 219**when you wanted to become pregnant for the last child? | 1. after __________months  2. the plan I had was not specific when to have a child | If they were pregnant with in their plan skip to Q 225 |
| 504 | If they were become pregnant before their plan, what were the main reasons for failing to postpone the last pregnancy? *(This question is indicated for those women who were become pregnant before their preferred time)* | 1. Lack of family planning use  2. I become pregnant while I were using modern family planning methods  3. I become pregnant while I were using traditional family planning methods  4. I become pregnant due to change of plan |  |
| 505 | What were the main reasons for failing to space the birth at least 33 months or 2 years before conception? *(This question is indicated for those women who were become pregnant before 24 months)* | 1. Lack of modern family planning use  2. I become pregnant while I were using modern family planning methods  3. I become pregnant while I were using traditional family planning methods  4. It was my plan to have a child at this time  5. others_________________________  (specify) |  |
| 506 | What were the main reasons to have optimum birth interval? *(This question is indicated for those women who have optimum birth interval)* | 1. To maintain my health  2. To insure economy security in the family  2. Others________________(specify) |  |

**Section III: Other environmental factors**

| **NO** | **Questions** | **Response** | **Skip** | |
| --- | --- | --- | --- | --- |
| 601 | Time taken to access the health institution from your home in minutes on foot? | ________minutes | |  |
| 602 | Source of information for reproductive health service utilization? | 1. Radio  2. TV  3. Newspaper  4. From health service providers  4. others______(specify) | |  |
| 603 | Who make a decision at home to use family planning methods? | 1.Husband  2.Self  2.Both  3.Relatives of the husband | |  |

**Thank you for your time!**
